# Invasive tree species affect terricolous bryophytes biomass and biodiversity in nutrient-poor but not nutrient-rich temperate forests

**DOI:** 10.1038/s41598-025-89917-x

**Published:** 2025-02-12

**Authors:** Sebastian Bury, Marcin K. Dyderski

**Affiliations:** Institute of Dendrology, Polish Academy of Sciences, Parkowa 5, 62-035 Kórnik, Poland

**Keywords:** Bryophyta, Invasion ecology, Aboveground biomass, Abundance gradients, Epigeic bryophytes, Mosses, Ecology, Biodiversity, Community ecology, Ecosystem ecology, Forest ecology, Forestry, Invasive species

## Abstract

Although invasive tree species pose a significant threat to biodiversity, their impact on terricolous bryophytes has been insufficiently recognized. In addition, almost all previous studies focused on differences between invaded and uninvaded stands, not accounting for the effect of invader abundance. Here we aimed to assess the impact of *Prunus serotina* and *Robinia pseudoacacia* on terricolous bryophytes on nutrient-poor (*Pinus sylvestris* stands) and nutrient-rich (*Quercus* spp. stands) sites, along a gradient of invader biomass. We assessed the cover and species richness of bryophytes by vegetation surveys in 160 study plots (100 m^2^) and biomass in eight 0.264 m^2^ circular subplots. Both tree species affected the cover and biomass of bryophytes, while *R. pseudoacacia* also reduced species richness. Although we did not confirm their impact on species composition except *R. pseudoacacia* in nutrient-poor sites, we revealed the negative individual responses of three species to invader biomass: *Pleurozium schreberi*, *Dicranum polysetum*, and *D. scoparium*. However, these impacts occurred only on nutrient-poor sites, but not on nutrient-rich sites. We provided the first quantitative assessment of *P. serotina* and *R. pseudoacacia* impacts on terricolous bryophytes along its biomass gradient. We showed low vulnerability of terricolous bryophytes in nutrient-rich broadlevaed sites while high in nutrient-poor coniferous sites, showing that there is no safe level of invader biomass.

**Supplementary Information:**

The online version contains supplementary material available at 10.1038/s41598-025-89917-x.

## Introduction

Biological invasions comprise one of the most important global drivers of biodiversity decline^1^. Among them, invasive tree species pose a particular threat due to their large dimensions, leading to dominance in community biomass and their long lifespan, maximizing the duration of impact on the ecosystem. Tree species, depending on their life-history traits, modify ecosystem functioning via the alteration of solar radiation transfer through the canopy^2,3^, leaf litter chemistry^4^, decomposition rates^5^, and in turn soil nutrients availability. For these reasons, invasive tree species can significantly modify invaded communities^6,7^. Indeed, alteration of ecosystem functioning by invasive tree species significantly affects various groups of dependent organisms, e.g. mycorrhizal fungi, bacteria, soil animals, or understory vegetation^2,8–10^.

Bryophytes are a group of plants especially significant for boreal and temperate forests, due to their contribution to understory primary production^11,12^. As their physiology depends on air humidity and they are strongly connected to particular substrates^13^, bryophytes are very sensitive bioindicators of changes in the ecosystem^14,15^. However, most studies on bryophyte diversity focus on well-preserved ecosystems, especially old-growth forests^16–18^. This trend is also reflected in ten times more studies assessing the impacts of non-native trees on vascular plants than on bryophytes^19^. Among 33 assessments of *P. serotina* effects on bryophytes only two revealed negative impact, four-positive impacts, and 27 showed no impact. For *Robinia pseudoacacia* none of the 17 analyzed cases revealed any impact of the invasive tree^19^. However, some studies, not included in the cited reviews, revealed more negative impacts of *P. serotina*^20,21^. Only a few studies assessed the effects of invasive tree species on plant biomass. Most of them focused on herbaceous plants^22^. Only two studies assessed the impact of invasive tree species on understory biomass. Bottollier-Curtet et al.^23^ found five times lower understory biomass beneath invasive *Acer negundo* canopy, compared to native *Salix alba* in France. López-Núñez et al.^24^ found that an increase in invasive *Acacia dealbata* cover from 0 to 100% decreased understory biomass by one order of magnitude in Portugal. However, none of them assessed the impact of invasive tree species on bryophyte biomass.

The vast majority of studies regarding the impact of invasive species on biodiversity focus on the comparison of invaded and uninvaded sites. This comparison allows to conclude about the maximum impact of invaders, but struggles to estimate the effect of invader quantity, which increases with invasion progress^24^. These contexts affect the impact of invaders, which usually is non-linear^25^. Therefore, assessment of impact per invader abundance unit can broaden our understanding of invasive species impacts on ecosystems^25,26^. Most studies assess these effects based on invasive species cover ^e.g. 27,28^. Only a few studies are based on more precise metrics, e.g. density or basal area^29^, or biomass^10,30,31^. However, up to now, only one study assessed the impact of invasive tree species abundance (expressed by density) on bryophytes^20^. The cited study confirmed the negative effect of increasing *P. serotina* density on terricolous bryophytes species richness.

We aimed to evaluate the impacts of two invasive tree species: *Prunus serotina* Ehrh. and *Robinia pseudoacacia* L. on terricolous bryophytes biomass, cover, and biodiversity in European temperate forests. These species are the most widespread in European woodlands^32^ and differ in life-history traits: maximum dimensions (larger *R. pseudoacacia* vs. smaller *P. serotina*), nitrogen-fixing ability (yes vs. no), and optimum successional stage (pioneer vs. mid-successional). We hypothesized that (H1) the increasing biomass of invasive tree species will impact terricolous bryophyte species composition, as well as decrease species richness, cover, and biomass. We also hypothesized that (H2) the impact of studied invasive tree species will differ between two environmental contexts: nutrient-rich *Quercus* spp. forests, which are somehow similar to ecological niche of studied species in their native range and nutrient-poor *P. sylvestris* forests, where studied invaders had been massively introduced^33–36^. We expected a lower impact of invasive tree species on nutrient-rich sites, where bryophytes comprise only a small part of understory biomass and species composition^37^ than in nutrient-poor forests.

## Results

**Fig. 1:**
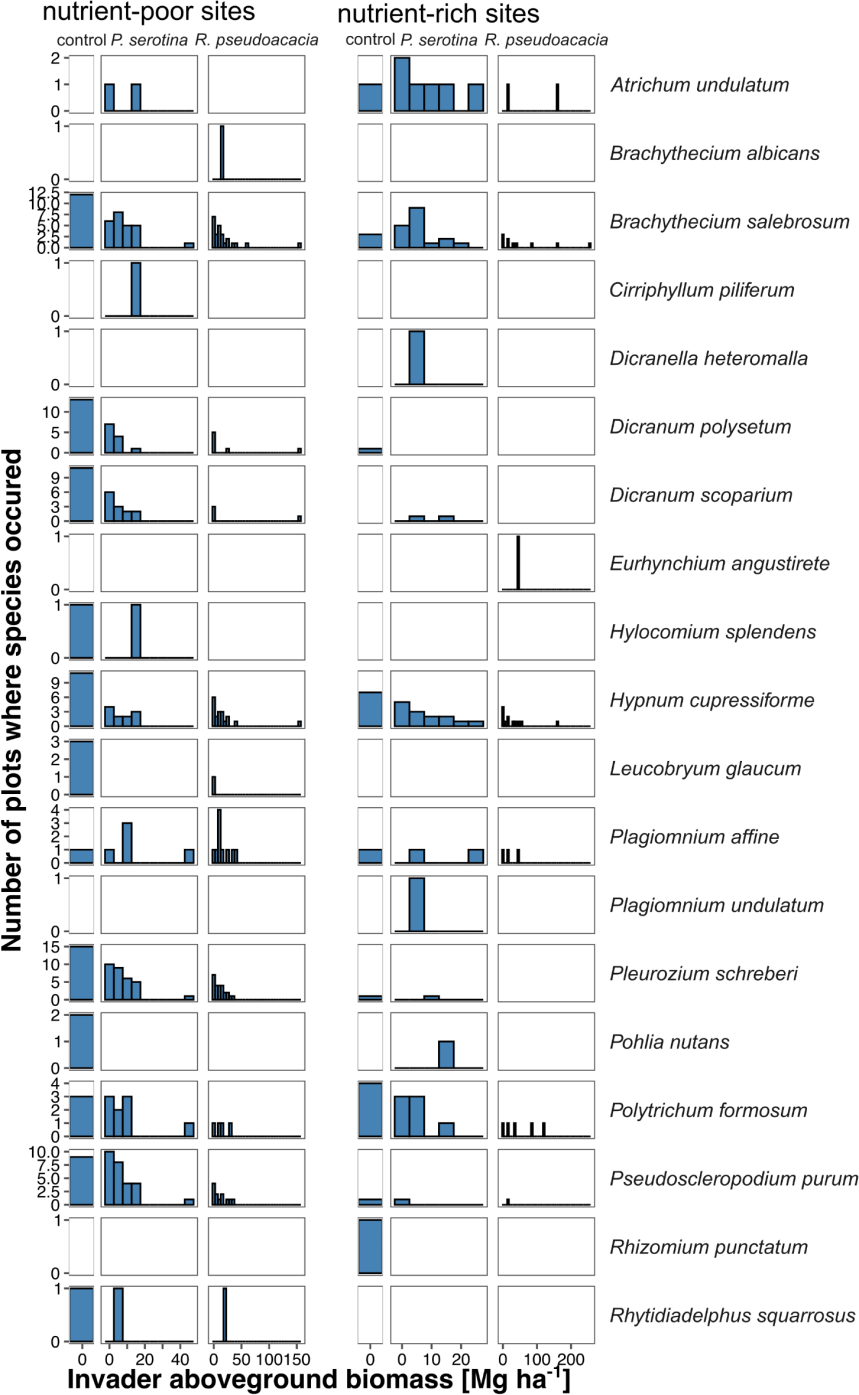
Distributions of all recorded bryophyte species occurrences along invasion gradients.

**Fig. 2:**
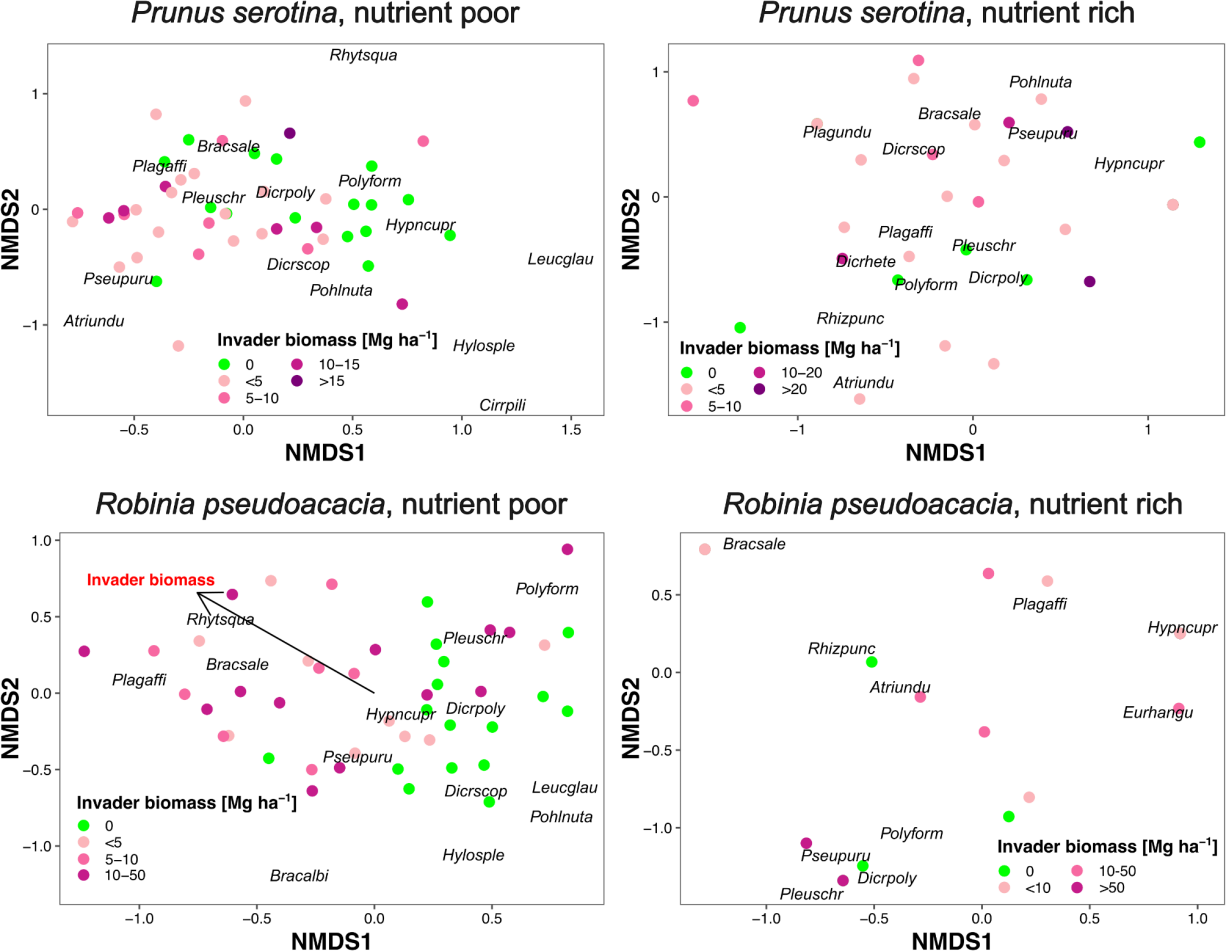
Results of Non-metric MultiDimensional Scaling of terricolous bryophyte species composition (*n* = 48 for *P. serotina* on nutrient-rich sites, *n* = 47 for *P. serotina* on nutrient-poor sites, and *n* = 45 for *R. pseudoacacia*). Study plots are represented by points (colored by invader biomass) while species – by abbreviated labels (four letters of genera name and species epithet, e.g. *Atriundu* = *Atrichum undulatum*). Correlation of invader biomass with NMDS axes was statistically insignificant (*p* > 0.05) in all cases except nutrient-poor *Robinia pseudoacacia* (Table S2).

We found 19 bryophyte species in 134 of the 160 study plots (Table S1). In the remaining 26 plots we did not find any bryophyte. Among them, the most frequent were *Brachythecium salebrosum* (occurring in 58.7% of the plots; Fig. 1), *Hypnum cupressiforme* (46.2%), *Pleurozium schreberi* (46.6%), *Pseudoscleropodium purum* (31.9%), *Dicranum polysetum* (20.6%), and *D. scoparium* (20.0%). Ordinations (NMDS) revealed a lack of invader biomass impact on bryophyte species composition in any habitat type and invasive species studied (*p* > 0.05; Fig. 2; Table S2), except nutrient-poor *R. pseudoacacia*. In nutrient-poor sites with *P. serotina* main gradient of species composition (NMDS1) reflected a transition from sites with a higher abundance of *P. schreberi* and *P. purum* toward sites with a higher abundance of *Leucobryum glaucum* and *H. cupressiforme*. The second axis (NMDS2), reflected the increasing abundance of *Rhitidiadelphus squarrosus*. In nutrient-rich sites with *P. serotina* NMDS1 axis showed a transition from sites with more *H. cupressiforme* towards those with more *Plagiomnium* spp. and *Rhizomnium punctatum*, while NMDS2 increased with increasing abundance of *Atrichum undulatum*. In nutrient-poor sites with *R. pseudoacacia* main gradient of species composition (NMDS1) reflected a transition from sites with a higher abundance of *B. salebrosum* and *P. affine* toward sites with a higher abundance of *Dicranum* spp. and *L. glaucum*. This gradient was opposite to the vector representing invader biomass. The second axis (NMDS2), reflected an increasing abundance of *B. albicans*. In nutrient-rich sites with *R. pseudoacacia*, both axes showed a blurred pattern.

**Fig. 3:**
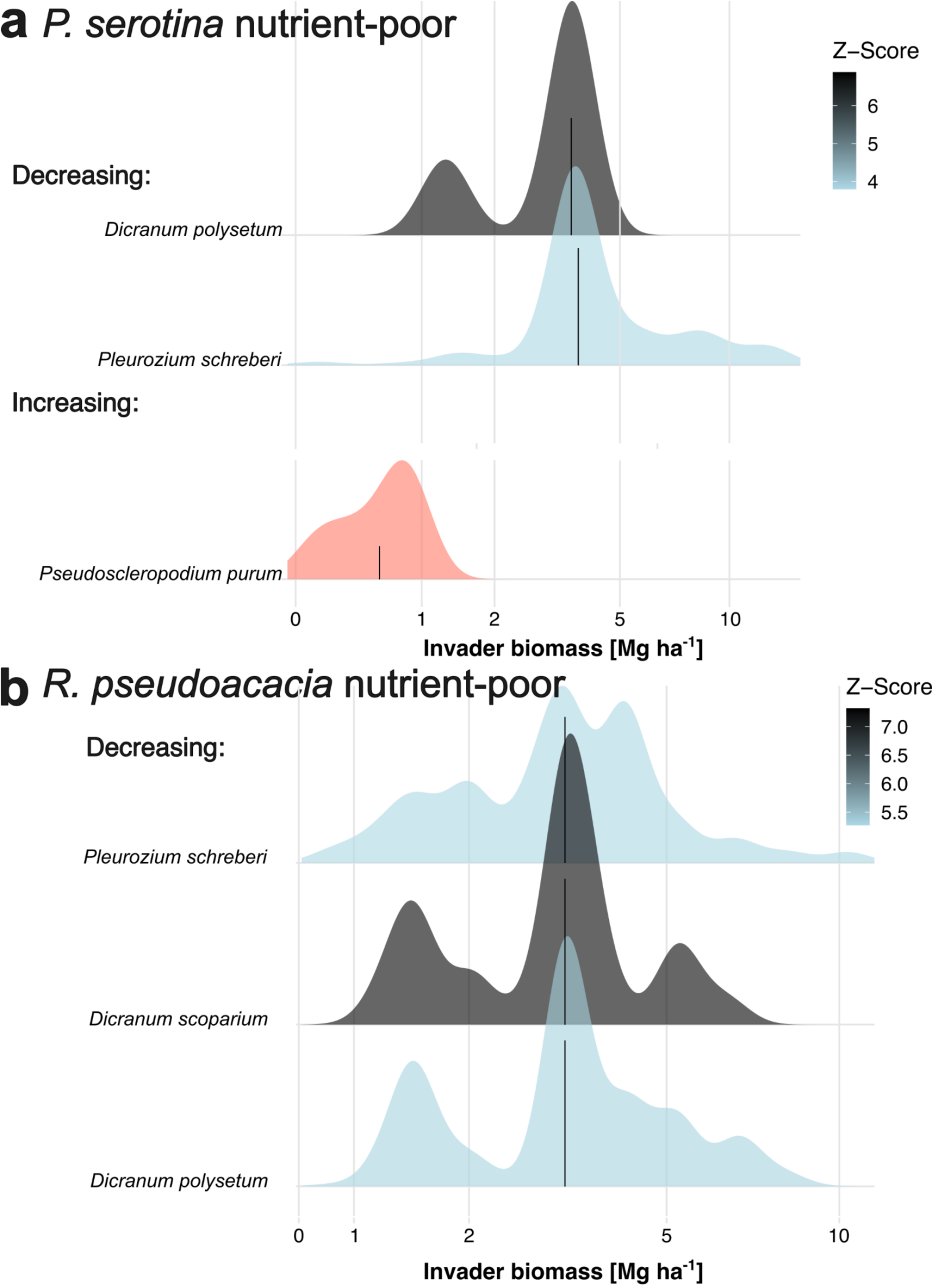
Results of Threshold Indicator Taxa Analysis for (**a**) nutrient-poor sites with *P. serotina* (*n* = 47 plots) and (**b**) nutrient-poor sites with *R. pseudoacacia* (*n* = 45 plots). Grey/blue density estimators represent species responding negatively to invader biomass gradient while red – those responding positively. Invader biomass was log(x + 1) transformed for analyses – note the logarithmic scale of horizontal axis. In the figure we presented only the species with reliability ≥ 0.95 and purity ≥ 0.95. For statistics of all species see Table S3.

The Threshold Indicator Taxa Analysis revealed the effect of invader biomass on the cover of some particular species (Fig. 3, Table S3). However, we found such impacts only in nutrient-poor habitats. An increase in *P. serotina* biomass above 0.8 Mg ha^− 1^ increased the cover of *P. purum*, while an increase over 3.8 Mg ha^− 1^ of *P. serotina* decreased the cover and frequency of *Dicranum polysetum* and *Pleurozium schreberi*. An increase in *R. pseudoacacia* biomass from 0 to 5 Mg ha^− 1^ slightly increased the cover and frequency of *P. schreberi*, *D. polysetum*, and *D. scoparium*, and then decreased their covers.

**Fig. 4:**
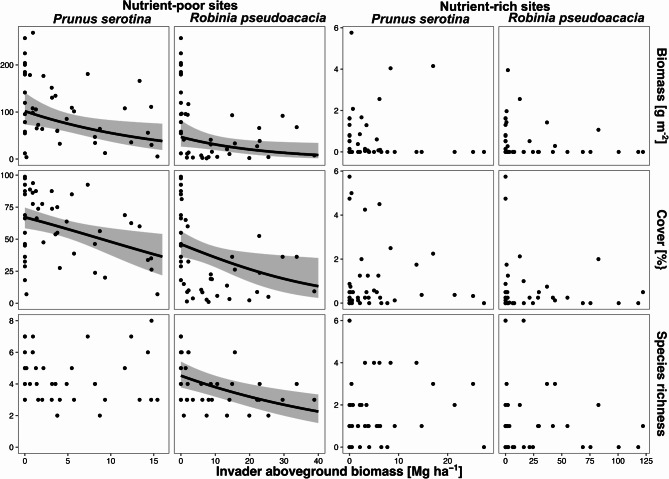
Marginal responses of bryophyte biomass, cover, and species richness to invader biomass, calculated from generalized linear mixed-effects models (Table 1). We provided regression lines only for models where the response to invader biomass was statistically significant (*p* < 0.05). Lines indicate marginal response (i.e. prediction without random effects and assuming all other predictors at a constant level), grey area—95% confidence intervals, and points—observed values (*n* = 48 for *P. serotina* on nutrient-rich sites, *n* = 47 for *P. serotina* on nutrient-poor sites, and *n* = 45 for both *R. pseudoacacia*).

Analysis of the impact of invader biomass on bryophyte species richness revealed various patterns between habitats and invasive species studied. The effect of invader biomass on bryophyte species richness was statistically significant only in the case of *R. pseudoacacia* on nutrient-poor sites (Fig. 4; Table 1). An increase in *R. pseudoacacia* biomass from 0 to 40 Mg ha^− 1^ reduced bryophyte species richness (from 4.5 ± 0.1 to 2.2 ± 0.2 species). On the other hand, bryophyte aboveground biomass clearly decreased with an increase in invader biomass, but only on nutrient-poor sites (Fig. 4; Table 1). On nutrient-poor sites, an increase in *P. serotina* biomass from 1 Mg ha^− 1^ to 10 Mg ha^− 1^ reduced bryophyte biomass by half (from 95.8 ± 0.2 g m^− 2^ to 55.3 ± 0.2 g m^− 2^), and further increase to 15 Mg ha^− 1^ reduced it down to 40.7 ± 0.3 g m^− 2^. An increase in *R. pseudoacacia* biomass from 0 to 40 Mg ha^− 1^ reduced bryophyte biomass by 80% (from 46.9 ± 0.3 g m^− 2^ to 8.6 ± 0.6 g m^− 2^). On nutrient-rich sites, the effect of invader biomass was statistically insignificant for both studied invasive tree species. We found a similar effect of studied invasive tree species on bryophyte cover: it was statistically significant only on nutrient-poor sites (Fig. 4; Table 1). An increase in *P. serotina* biomass from 1 Mg ha^− 1^ to 10 Mg ha^− 1^ decreased bryophyte cover from 63.5 ± 17.1% to 47.9 ± 21.9%, and a further increase in invader biomass to 15 Mg ha^− 1^ reduced it down to 38.2 ± 34.0%. An increase in *R. pseudoacacia* biomass from 0 to 40 Mg ha^− 1^ reduced bryophyte cover by 70% (from 46.1 ± 20.9% to 13.3 ± 64.9%)^1^.

**Table 1:** Generalized linear mixed-effects models for bryophyte species richness, cover (%), and biomass (g m^− 2^). The family of distribution used for each model is provided below the habitat type and invader classes (*n* = 48 for *P. serotina* on nutrient-rich sites, *n* = 47 for *P. serotina* on nutrient-poor sites, and *n* = 45 for both *R. pseudoacacia*).

Habitat type and invader classes/(distribution)	Variable	Estimate	SE	z	Pr(>|z|)	RE	SD	R^2^
Response = species richness								
*P. serotina* poor sites (COM-Poisson)	(Intercept)	1.6041	0.1547	10.366	< 0.001	Insp.	< 0.0001	R^2^_m_ = 0.002
	Invader biomass	-0.0060	0.0095	-0.629	0.530	Year	0.2415	R^2^_c_ = 0.146
*P. serotina* rich sites (Poisson)	(Intercept)	0.2895	0.2431	1.191	0.234	Insp.	0.1919	R^2^_m_ = 0.019
	Invader biomass	0.0205	0.0195	1.049	0.294	Year	0.0854	R^2^_c_ = 0.064
*R. pseudoacacia* poor sites (COM-Poisson)	(Intercept)	1.5088	0.0921	16.378	< 0.001	Insp.	< 0.0001	R^2^_m_ = 0.079
	Invader biomass	-0.0174	0.0053	-3.279	**0.001**	Year	0.1012	R^2^_c_ = 0.104
*R. pseudoacacia* rich sites (negative binomial)	(Intercept)	0.1486	0.2687	0.553	0.580	Insp.	0.1681	R^2^_m_ = 0.002
	Invader biomass	-0.0031	0.0058	-0.536	0.592	Year	< 0.0001	R^2^_c_ = 0.001
Response = cover								
*P. serotina* poor sites (Beta)	(Intercept)	0.7124	0.1888	3.772	0.000	Insp.	< 0.0001	–
	Invader biomass	− 0.0795	0.0289	− 2.748	**0.006**	Year	< 0.0001	–
*P. serotina* rich sites (ZI Beta)	(Intercept)	− 4.4469	0.3008	− 14.785	< 0.001	Insp.	0.3063	–
	Invader biomass	0.0120	0.0272	0.443	0.658	Year	< 0.0001	–
	ZI: (Intercept)	− 0.8834	0.3884	− 2.275	0.023			–
	ZI: Invader biomass	− 0.0255	0.0540	− 0.473	0.636			–
*R. pseudoacacia* poor sites (ZI Beta)	(Intercept)	− 0.1562	0.2091	− 0.747	0.455	Insp.	0.0026	–
	Invader biomass	− 0.0430	0.0188	− 2.293	0.022	Year	0.0020	–
	ZI: (Intercept)	− 12.7130	142.6720	− 0.089	0.929			–
	ZI: Invader biomass	− 2.9170	1353.6330	− 0.002	0.998			–
*R. pseudoacacia* rich sites (ZI Beta)	(Intercept)	− 4.6206	0.2299	− 20.102	< 0.001	Insp.	0.0012	–
	Invader biomass	− 0.0004	0.0057	− 0.072	0.942	Year	0.0013	–
	ZI: (Intercept)	− 0.5044	0.3625	− 1.392	0.164			–
	ZI: Invader biomass	0.0047	0.0091	0.512	0.608			–
Response = biomass								
*P. serotina* poor sites (Gaussian)	(Intercept)	4.6324	0.1602	28.910	< 0.001	Insp.	< 0.0001	R^2^_m_ = 0.108
	Invader biomass	− 0.0601	0.0254	− 2.370	**0.022**	Year	< 0.0001	R^2^_c_ = 0.108
*P. serotina* rich sites (Gaussian)	(Intercept)	0.2681	0.1644	1.631	0.269	Insp.	< 0.0001	R^2^_m_ = 0.004
	Invader biomass	0.0052	0.0124	0.421	0.676	Year	0.2242	R^2^_c_ = 0.171
*R. pseudoacacia* poor sites (Gaussian)	(Intercept)	3.8685	0.2749	14.072	< 0.001	Insp.	0.2194	R^2^_m_ = 0.092
	Invader biomass	− 0.0402	0.0189	− 2.123	**0.040**	Year	< 0.0001	R^2^_c_ = 0.117
*R. pseudoacacia* rich sites (Gaussian)	(Intercept)	0.2999	0.1294	2.317	0.121	Insp.	< 0.0001	R^2^_m_ = 0.028
	Invader biomass	− 0.0022	0.0018	− 1.223	0.228	Year	0.1890	R^2^_c_ = 0.213

*SE* standard error, *z/t* test statistic (t for biomass models with Gaussian distribution, z for species richness and cover), *p* p-value, *RE* random effects (Insp.—forest inspectorate), *SD* standard deviation of random effect, R^2^_m_ marginal coefficient of determination (amount of variance explained by fixed-effects only), R^2^_c_ conditional coefficient of determination (amount of variance explained by both fixed and random effects). We did not provide R^2^_m_ and R^2^_c_ for Beta models as for this distribution calculations provide unreliable values (> 1.0).

## Discussion

Our study partially confirmed the first research hypothesis (H1). We found an impact of the studied invasive tree species mostly on cover and biomass of bryophytes. We did not confirm the effect on species composition, except *R. pseudoacacia* in nutrient-poor sites. We revealed the individual responses of three species to the increased biomass of invasive tree species. However, our study confirmed the second hypothesis (H2), we showed that these impacts of invasive tree species occurred only in Scots pine forests.

### Impact of invasive tree species: differences between Scots pine and oak forests

We found that the studied invaders affected terricolous bryophytes only on nutrient-poor sites, where bryophytes comprised a significant part of the understory vegetation^37–39^. Although maximum bryophyte species richness was quite similar in both nutrient-poor and nutrient-rich sites, biomass and cover were 20 times lower, due to a lower importance of bryophyte for shaping broadleaved forests understory. In both types of sites the pool of bryophyte species covered both species typical of particular forest type and generalists, with wide ecological requirements. For that reason, we observed higher impacts in sites where bryophytes were important elements of the understory. However, the observed differences are related both to soil fertility and tree species effect. As most of nutrient-poor forest types in Poland are coniferous, and most nutrient-rich are deciduous, it is hard to separate soil fertility effect from canopy characteristics, related to species-specific light transmittance and nutrient cycling dynamics. Using our dataset we can say that invasive species do not affect nutrient-rich sites, only considering those with a broadleaved canopy. For that reason our conclusions are limited to comparison between nutrient-poor coniferous forests and nutrient-rich broadleaved forests.

### Impact of ***Prunus serotina*** on terricolous bryophytes

Our study extends the conclusions from a previous study, comparing terricolous bryophytes cover and richness in nutrient-poor *P. sylvestris* forest in Wielkopolska National Park^40^. In the cited study, the authors found a higher species richness and a lower cover in invaded compared to non-invaded plots. Here we confirmed the impact of the invaders on the cover, with a linear decrease in cover along the invader biomass gradient, but we did not find this effect on species richness. This result is also different from other studies. For example, Verheyen et al.^20^ revealed a negative effect of *P. serotina* density on bryophyte species richness. Halarewicz and Pruchniewicz^21^ resampled nutrient-poor *P. sylvestris* forest understory vegetation after ten years, where the canopy cover of *P. serotina* increased ten times. They found a decrease in bryophyte species richness by 60% and in bryophyte cover by 85%. The difference between the cited study and our results in species richness may be related to the size of the species pool. It can be also related to other metrics of invader quantity, e.g. density^27^, which is less correlated with biomass than other variables^31^. The mean species richness in the cited study for the historical dataset was twice as low as in our study. That way, the bryophyte community was more vulnerable to the loss of particular species. Our study, similar to the cited one, found *P. schreberi* to be the most sensitive to *P. serotina*. However, we found that *D. polysetum* negatively responded to *P. serotina* while Halarewicz and Pruchniewicz^21^ found *D. scoparium* more sensitive than *D. polysetum*. They also did not record *P. purum* which in our study increased its frequency with increasing *P. serotina* biomass. This bryophyte has slightly higher soil fertility requirements (3 vs. 2 for *P. schreberi* and *D. polysetum*) and much higher soil reaction requirements (6 vs. 2 for *P. schreberi* and 3 for *D. polysetum*), according to ecological indicator values^41^. Different response of *P. purum* suggests the possible replacement of *P. schreberi* and *D. polysetum* by *P. purum*, leading to a lack of response in species richness. Another explanation can be related to the preference of *P. schreberi* and *D. polysetum* towards *P. sylvestris* forests older than 40 years. In younger stands light availability decreases up to reaching the maximum canopy closure, and after a few years since that time light availability increases, due to self-thinning favoring the mentioned bryophyte species^39^. With increasing biomass of *P. serotina*, light availability decreases negatively affecting the cover of *P. schreberi* and *D. polysetum*^20,42^.

The mechanisms of negative *P. serotina* impact on terricolous bryophytes might be related to decreasing light availability and increasing soil fertility. *P. serotina* can intercept significant amounts of solar radiation transmitted through the canopy^3,42^. Thus, it changes the conditions in the forest floor layer, in *P. sylvestris* forest dominated by light-demanding dwarf shrubs, grasses, and bryophytes^37,39^. Moreover, due to higher leaf litter nutrient content and decomposition rate, compared to *P. sylvestris*^4,5^, it increases the available pool of nutrients. These two transformations decrease the level of resource limitation, allowing for the encroachment of more competitive species, usually with lower light and higher nutrient requirements^37,41^.

### Impact of ***Robinia pseudoacacia*** on terricolous bryophytes

Our results indicating the lack of *R. pseudoacacia* effect on terricolous bryophytes on nutrient-rich sites align with our previous study^40^, where we found no differences in species richness and cover between *R. pseudoacacia* forests and a wide range of studied forest types, from nutrient-poor *P. sylvestris* forests to *Quercus-Carpinus-Tilia* nutrient-rich forests. However, *R. pseudoacacia* stands were only on nutrient-rich sites and, therefore cannot be compared with nutrient-poor *P. sylvestris* forests. Although *R. pseudoacacia* was planted in nutrient-poor sites^34,35,43^, most of the studies assessing its impact on vegetation focused on nutrient-rich sites^8,44^. In these communities bryophytes usually comprise a small part of understory vegetation and, thus were not assessed. On nutrient-poor sites we found a decrease in species richness, cover, and biomass along the *R. pseudoacacia* biomass gradient. We also found a shift in species composition, from species typical of coniferous forests (e.g. *D. scoparium* or *Leucobryum glaucum*) toward generalists (e.g. *Rhytidiadelphus squarrosus* or *Brachythecium salebrosum*). The decrease in species richness was stronger than in the case of *P. serotina*. The mechanisms of impact could be similar to those of *P. serotina*: limitation of light availability and increase in soil fertility. However, larger decreases of bryophytes might be connected with higher biomass reached by *R. pseudoacacia*. This species increases the soil nitrogen pool^5,44^, supporting the encroachment of more competitive species that can compete with bryophytes typical of coniferous forests. Although *R. pseudoacacia* does not intercept much more light than native species per basal area unit^3^, light availability in stands with this species is usually low^40,44^. This can result from enhancing the understory, intercepting more light, usually by nitrophilous shrubs and tall herbs^8,43,44^. The whole-community response was confirmed by individual responses of three species comprising a significant part of the understory and decreasing with invader biomass: *P. schreberi*, *D. polysetum*, and *D. scoparium*.

### Management and conservation implications

Our study provided a step forward in understanding the impacts of invasive tree species on terricolous bryophytes. On nutrient-rich sites with broadleaved canopy, we did not record any impact of *P. serotina* or *R. pseudoacacia* on terricolous bryophytes. This suggests that for bryophytes there are no concerns regarding studied invasive tree species in nutrient-rich sites with broadleaved canopy, and more emphasis should be put on the assessment of their impact on overall ecosystem functioning and other groups of organisms, that can be more affected. This information is especially important for case-specific management, accounting for wide environmental and economic contexts of *P. serotina*^36^ and *R. pseudoacacia*^35,43^. However, from the overall conservation point of view, diluting the proportion of studied invaders in the forest by native species can result in more stable and less risky mixed stands^35^. Diluting might be achieved by supporting the growth of native competitors and careful, partial removal of studied invaders.

On nutrient-poor sites, we confirmed the abundance-dependent threat that the invasive tree species represent to terricolous bryophytes. Even small biomass of studied invaders resulted in decreased cover, biomass, and—in the case of *R. pseudoacacia*—species richness. That way, terricolous bryophytes of *P. sylvestris* forests on nutrient-poor sites are especially vulnerable to studied invaders, and there is no safe level of invader biomass. Reducing the biomass of studied invaders might involve either direct eradication measures or thinning. The former requires time and labor, and in some cases, application of herbicides might be necessary to avoid resprouting after cutting. However, in some countries use of herbicides is not allowed, especially in nature conservation areas. Moreover, most of the eradication efforts fail due to the high vegetative reproduction potential of the studied species and the necessity of control for resprouts and root suckers over a few years after cutting^35,36^. For that reason removing the studied invaders can lead to their further spread. Therefore, increasing forest resistance to invasion should focus on maintaining canopy closure, as studied invaders benefit from increased light availability^33,34,42,43,45^. That way, maintaining a low invasibility of forest patches (by keeping the canopy closed) becomes at least as important as eradication, and easier to introduce in management practice.

## Methods

### Studied invasive species

Both studied invasive species originally occurred in North America and were initially planted in Europe as utility and ornamental trees. Due to massive plantations, they successfully spread into surrounding forests and nowadays they are among the most widespread invasive tree species in Central Europe^33,34,46^. Differences in the ecology of studied species affect their different impacts on ecosystems^19,46,47^. *Robinia pseudoacacia* from the Fabaceae family has nitrogen-fixing ability and enriches the soil by its leaf decomposition^48,49^. Thus, *R. pseudoacacia* can grow in even very poor habitats. It has its light availability optimum in open sites, as a typical pioneer species^34^. Another important feature of the species affecting its invasiveness and difficulty in eradicating is the ability to spread via root suckers^50^. *Prunus serotina* grows fast and produces many fruits, successfully spread by birds and mammals, but also falling beneath the canopy of parental trees^51–53^. This species has a very wide range of ecological optimum, but prefers occurrence in canopy gaps^42,54,55^. In Central Europe it usually grows as a low tree or shrub^34,47^. Unlike *R. pseudoacacia*, it cannot fix nitrogen, but also affects soil biogeochemical cycles, enriching soil in nutrients^4,5^. In the study area, both *P. serotina* and *R. pseudoacacia* were planted in the past (till the 1980s) as admixtures aiming to enrich soil nutrients and support the biodiversity of animals. These species massively spread into surrounding forests^56,57^. In 56 of 57 bored *P. serotina* trees, we found the oldest radial increments from the 1990s, while in 40 of 60 *R. pseudoacacia*—from the 1980s (Muzolf et al. *in prep*), i.e. most of the trees started growth after these species become no longer planted in forests.

### Study design

**Fig. 5:**
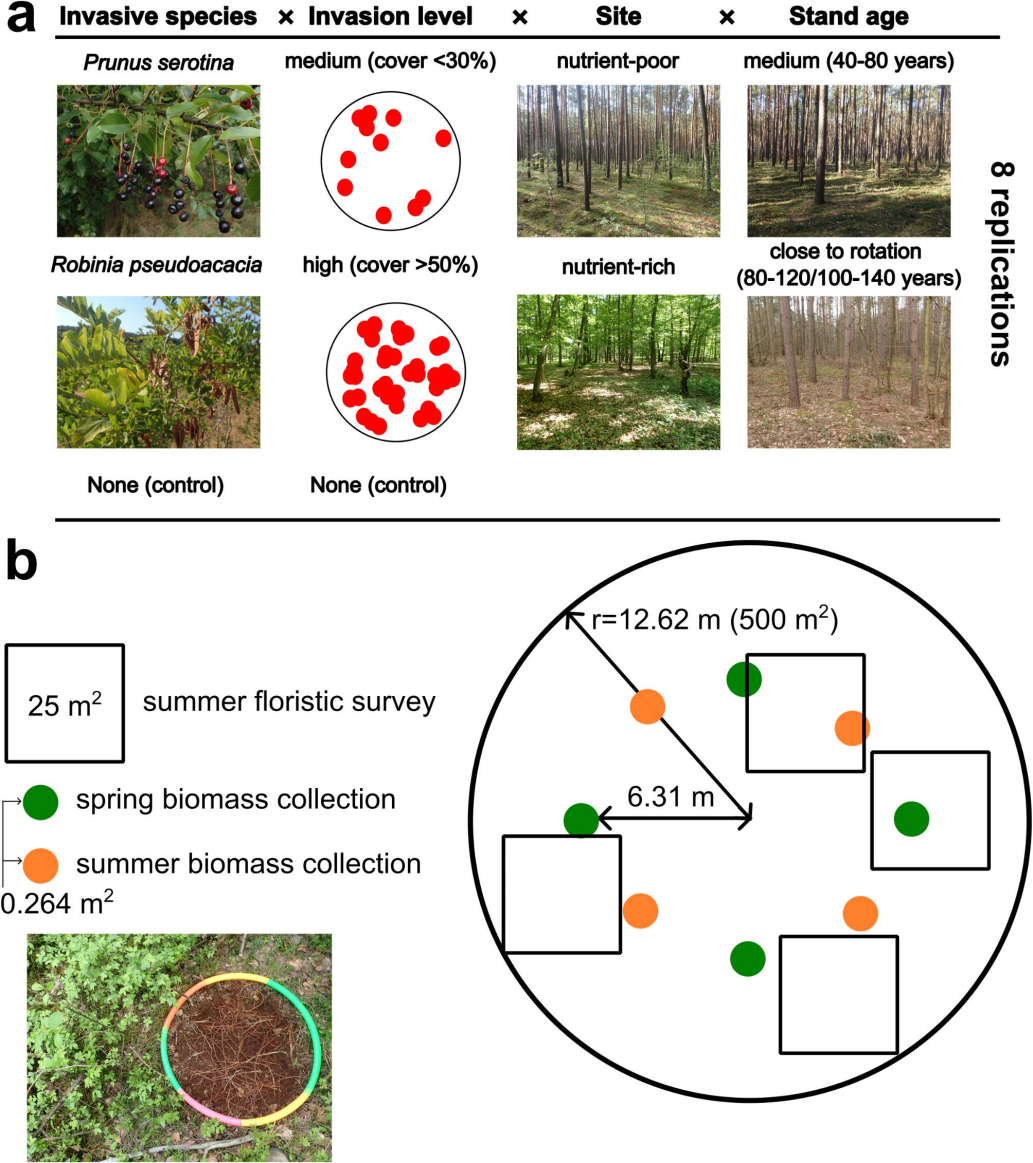
Above (**a**): Scheme of the study design showing invasive species studied, levels of invasion used for plot selection, site types, and stand ages. Below (**b**): scheme of study plot and subplots with the photo of one subplot (0.264 m^2^) after collection of all understory components.

We conducted the study in managed forests of Western Poland (Fig. S1), characterized by the climate typical of a temperate zone, transitional between maritime and continental. The mean annual temperature in the study area was 8.5 °C while mean annual precipitation ranged between 500 and 550 mm. To cover two environmental contexts, we selected study plots (500 m^2^) on two forest types: nutrient-poor sites with *Pinus sylvestris* and nutrient-rich with *Quercus* spp. (*Q. robur* and *Q. petraea*; Fig. 5a). We selected them using the national forest database forest habitats classification, which is related to the main types of forest vegetation. For nutrient-poor sites, we selected habitats described as ‘fresh mixed-coniferous forests’, which in that classification represent nutrient-poor, acidophilous soils and mainly *P. sylvestris* forests in lowlands. We narrowed the scope of plot searching to stands with *P. sylvestris* domination, avoiding acidophilous oak forests, to not introduce another driver of variability. Similarly, as nutrient-rich sites, we choose habitats described as ‘fresh mixed-broadleaved forests’ or ‘fresh broadleaved forests’, which represent mesic to fertile broadleaved forests, mostly oak or beech forests. We narrowed it down to oak-dominated forests. Nutrient-poor sites usually grew on rustic soils or podzols and were covered by suboceanic coniferous forest *Leucobryo-Pinetum* or by secondary communities with *P. sylvestris*. Nutrient-rich sites usually occurred on luvisols, leptosols, or cambic soils, covered by oak-hornbeam forest *Galio sylvatici-Carpinetum* or secondary oak forests^47^. We selected study plots in two age classes: near rotation age (80–120 years for *P. sylvestris* and 100–140 years for *Quercus* spp.) and in the middle of rotation age (40–80 years). We selected sites similar in terms of management (even-aged, not thinned in the last few years, originating from planting), trying to maintain a similar level of management impact. The stand age on our plots varied from 42 to 139 years old for *Quercus* spp. stands and from 42 to 117 years old for *P. sylvestris* stands. We aimed to cover the gradient of invasive species biomass. We decided to designate control plots per habitat type and age class (n = 8, in total n = 32; Fig. 5a), without the presence of the studied invasive tree species in shrub and canopy layers (ensuring lack of studied invasive species higher than > 1.3 m). For each studied invasive tree species, age class, and habitat we searched eight plots with medium invader abundance (initially assessed by canopy cover < 30%), and eight with a high abundance (> 50%), to ensure a gradient of invasive tree species biomass across plots. We selected only plots with homogenous understory vegetation and canopy cover, maintaining a distance of 5 km between plots of the same habitat type and age class, to avoid spatial autocorrelation. After a field check of all criteria, we finally obtained a set of 160 study plots (Fig. S1).

### Invasion gradient

**Fig. 6:**
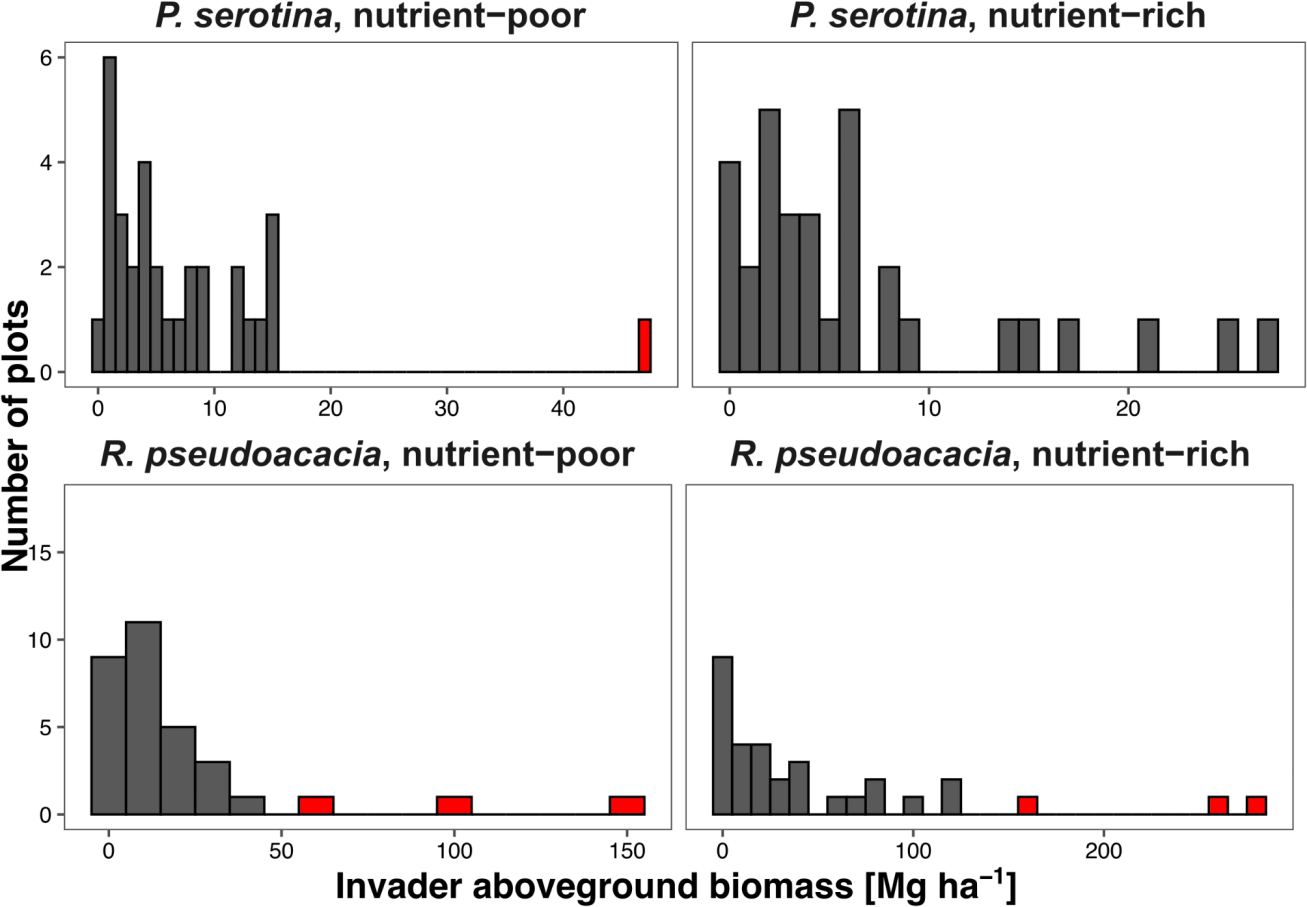
Histograms visualizing the distribution of invasive tree aboveground biomass within study plots (*n* = 32 per habitat type and invader classes). For all control plots (*n* = 32 for nutrient-rich and *n* = 32 for nutrient-poor sites) invader aboveground biomass was of 0.00 Mg ha^− 1^. Outlier excluded from analyses were marked by red color.

Within each study plot, for each species, we measured the diameter at the breast height of all trees over 1.3 m height. We used these measurements for calculating the aboveground biomass of studied invasive tree species, using published and unpublished allometric models (Table S4)^58–60^. We decided to use aboveground biomass as a metric accounting not only for the size of stems, but also for tissue density and the amount of resources used for development^61^. Invasion gradient was strongly correlated with basal area, proportion of invader aboveground biomass to total stand aboveground biomass, and proportion of invader basal area to total stand basal area in each pair of studied invasive species and habitat type (Pearsons’s r ranging from 0.878 to 0.993; Table S5; Fig. S2). The invasion gradient in the stand with *P. serotina* on nutrient-poor sites ranged from 0.18 to 47.11 Mg ha^− 1^, with an average of 7.34 ± 1.55 Mg ha^− 1^ (Fig. 6). We excluded the last observation, which was three times higher than the second highest result, and acted as an outlier in models. After that, it ranged from 0.18 to 15.43 Mg ha^− 1^, with an average ± SE of 6.05 ± 0.89 Mg ha^− 1^. On nutrient-rich sites it ranged from 0.18 to 27.39 Mg ha^− 1^, with an average of 6.68 ± 1.28 Mg ha^− 1^. Invasion gradient in the stand with *R. pseudoacacia* on nutrient-poor sites ranged from 0.22 to 153.00 Mg ha^− 1^, with an average of 20.91 ± 5.60 Mg ha^− 1^, while on nutrient-rich sites it ranged from 0.82 to 278.24 Mg ha^− 1^, with an average of 50.77 ± 12.44 Mg ha^− 1^. For a similar reason we excluded three plots for *R. pseudoacacia* both for nutrient-poor sites (*R. pseudoacacia* aboveground biomasses = 153.00 Mg ha^− 1^, 104.05 Mg ha^− 1^, 57.92 Mg ha^− 1^) and nutrient-rich (*R. pseudoacacia* aboveground biomasses = 159.35 Mg ha^− 1^, 255.43 Mg ha^− 1^, 278.24 Mg ha^− 1^). After that, the invasion gradient in the stand with *R. pseudoacacia* on nutrient-poor sites ranged from 0.22 to 38.83 Mg ha^− 1^, with an average of 12.21 ± 2.27 Mg ha^− 1^, while on nutrient-rich sites it ranged from 0.82 to 122.16 Mg ha^− 1^, with an average of 32.12 ± 5.96 Mg ha^− 1^.

### Bryophyte survey

In each plot, we established four square subplots (25 m^2^ each), randomly distributed within a plot (Fig. 5b). Within subplots, we surveyed terricolous bryophytes using a modified, nine-degree Braun-Blanquet scale^62^, i.e. adding three categories of cover in the most frequent class (‘2’ in original Braun-Blanquet scale split into 2 m, 2a, and 2b, representing < 5%, 5–15%, and > 15%, respectively). We surveyed only terricolous bryophytes, i.e. only those occurring on the soil, excluding epiphytic, epilithic, and epixylic bryophytes to compare our results with previous studies, e.g. ^21,40^. We surveyed bryophyte species composition in summer 2021, 2022, and 2023. In 2021 we sampled 47 plots, in 2022 67, and in 2023 46 plots. We collected four samples of bryophyte aboveground biomass in spring and four in summer, in sampling spots not affected by trampling during other research activities within plots (Fig. 4b). Within each plot, we systematically established four sampling spots, according to the cardinal direction in spring (north, east, south, and west), and we rotated this by 45° in summer (northeast, southeast, southwest, and northwest; Fig. 5b). We set sampling spots 6.31 m away from the plot center (midpoint of the main plot radius), using a compass and measuring tape. In each of them, we placed a circular frame (d = 58 cm, i.e., 0.264 m^2^) and we collected all plants within a frame, separating it into herbaceous plants and bryophytes. After that we transported material to the lab and using tweezers we cleaned bryophyte samples from vascular plants, animals, and litter. Then, we sieved samples to separate sand and small particles of litter. After cleaning, we dried the material up to a constant mass in the oven (65 °C) and we weighed it with an accuracy of 0.001 g. We decided to sample on two seasons, as we simultaneously sampled understory herbaceous plants that differed in biomass production patterns between spring and summer^63^. We decided to include data from both seasons and average it, as our sampling unit for biomass (0.264 m^2^) was smaller than vegetation survey plots (25 m^2^), to increase the stability of the results.

### Data analyses

We analyzed data using R software, version 4.3.2^64^. Mean values are followed by ± standard error (SE). For each study plot, we averaged the cover of each bryophyte species over the four squared subplots. We conducted all analyses separately for each studied invasive species and habitat type. This resulted in *n* = 48 in each habitat type and invader class, except *P. serotina* on nutrient-poor sites, where we excluded a single outlier (Fig. 6), where *P. serotina* aboveground biomass was 47.11 Mg ha^− 1^, as this value exceeded by three times the second-highest value (15.43 Mg ha^− 1^). For *R. pseudoacacia* we excluded three extremal plots for both nutrient-poor (*R. pseudoacacia* aboveground biomasses = 153.00 Mg ha^− 1^, 104.05 Mg ha^− 1^, 57.92 Mg ha^− 1^) and nutrient-rich sites (*R. pseudoacacia* aboveground biomasses = 159.35 Mg ha^− 1^, 255.43 Mg ha^− 1^, 278.24 Mg ha^− 1^) (Fig. 6).

We analyzed the species composition of bryophytes using Non-Metric Multidimensional Scaling (NMDS), implemented in the vegan package^65^. We decided to use unconstrained analysis due to long gradients of species composition, resulting in artifacts when we conducted preliminary constrained analyses (redundancy analysis or canonical correspondence analysis). We tested a passive fitness of invader biomass (log1p-transformed) using the envfit() function from the vegan package^65^, to see whether this variable is correlated with the main gradients of bryophyte species composition.

We analyzed the responses of particular bryophyte species to invader biomass using the Threshold Indicator Taxa Analysis, implemented in the TITAN2 package^66^. This analysis assesses the cover and frequency of studied species along environmental gradient and was previously used e.g. to detect the impact of non-native *Pseudotsuga menziesii* on understory plant species^67^. The main advantage of this tool is not only the formal selection of species increasing and decreasing along the gradient but also the indication of species optima. In this analysis we stabilized the dependent variable (invader biomass) using log(x + 1) transformation.

We assessed the impact of invader aboveground biomass on bryophytes species richness, cover, and aboveground biomass using Generalized Linear Mixed-Effects (GLMMs) models, implemented in the glmmTMB package^68^. For species richness, as a count variable, we assumed the Poisson distribution of the dependent variable. We checked the dispersion of Poisson GLMMs using the formal test implemented in the DHARMa package^69^. For three habitat types and invader classes we did not find statistically significant over- or under-dispersion, thus we used Poisson GLMMs as final models. For *P. serotina* on nutrient-poor sites we found statistically significant under-dispersion, therefore we used COM-Poisson GLMM^70^. For bryophyte cover we used zero-inflated Beta GLMMs, designed for proportional data. We assumed the normal distribution of the dependent variable for aboveground biomass, but due to high dispersion in data we used log1p transformation, i.e., natural logarithm of x + 1. This approach allowed us to include zero values in the analyses. In all models we used invader biomass as a fixed effect while year of investigation and forest inspectorates (units of forest divisions) as random intercepts. These random effects covered spatial (forest inspectorate) and temporal (year) dependence among samples. For GLMMs we reported values of two coefficients of determination: marginal (R^2^_m_) and conditional (R^2^_c_), that inform about the amount of variance explained by fixed effects only, and both fixed and random effects, respectively^71^. As for Beta models values of R^2^_m_ and R^2^_c_ are unreliable (exceeding 1.0), we did not provide them. We visualized the results using marginal responses, i.e. mean predicted response to each level of a particular predictor, assuming all remaining predictors at a constant level and excluding random effects (global population estimate). We calculated them using the ggeffects package^72^.

## Electronic supplementary material

Below is the link to the electronic supplementary material.

Supplementary Material 1

## Data Availability

All data supporting the results are archived in the figshare repository DOI: 10.6084/m9.figshare.26842303. For review purposes use the link https://figshare.com/s/1a7be2791ad9e37dfe8d.
