# Supplementary material for: Invasive tree species affect terricolous bryophytes biomass and biodiversity in nutrient-poor but not nutrient-rich temperate forests
Source: Sci Rep. 2025 Feb 12;15:5272. doi: 10.1038/s41598-025-89917-x (PMC11822004; doi:10.1038/s41598-025-89917-x)
Supplement: Supplementary file 1 — Supplementary Material 1 [file 41598_2025_89917_MOESM1_ESM.docx]

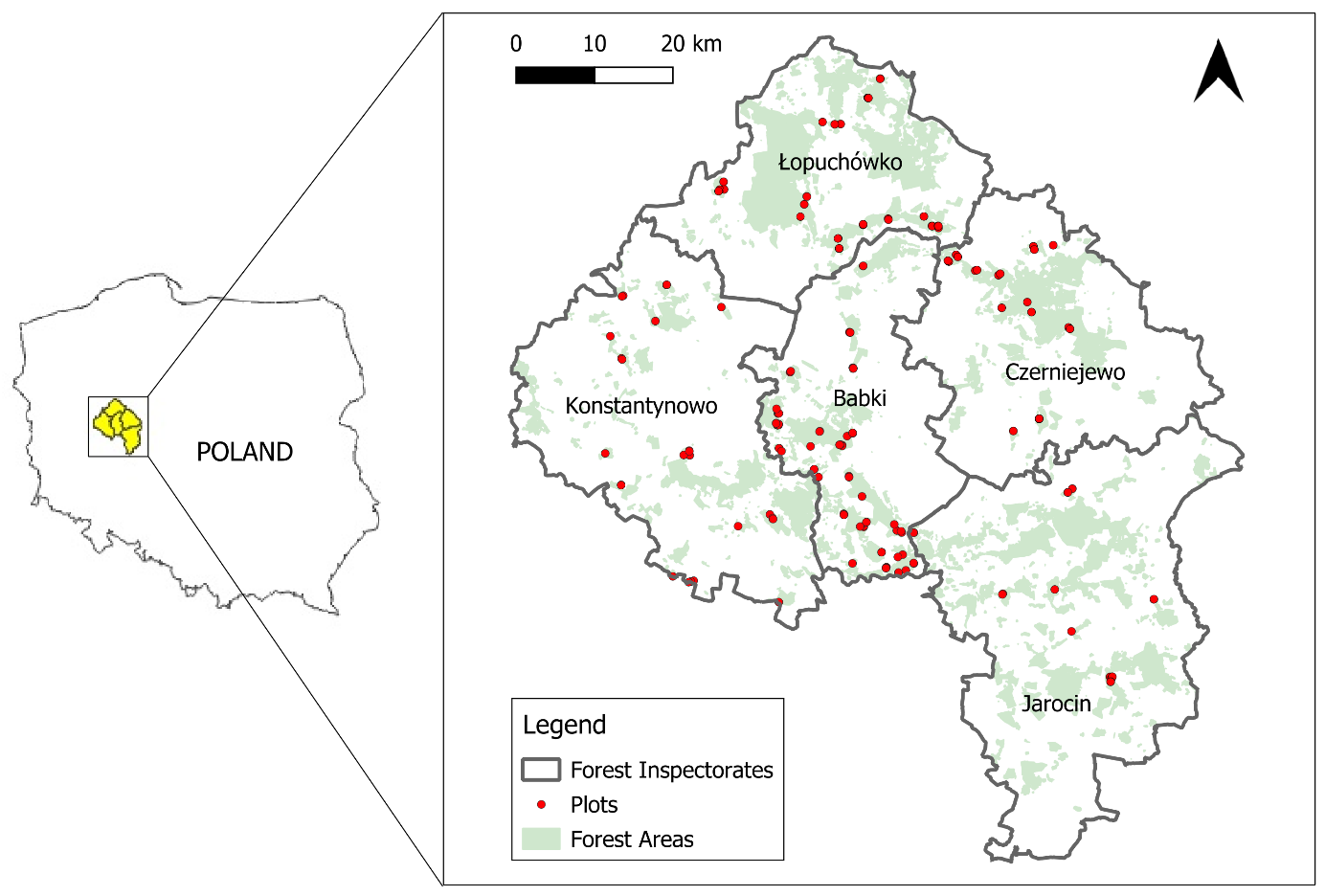


**Fig. S1.** Map of the study plots (n=160) on the background of forest cover and borders of forest inspectorates, obtained from the Forest Data Bank ([www.bdl.lasy.gov.pl/portal/mapy](http://www.bdl.lasy.gov.pl/portal/mapy)). Reproduced from Bury and Dyderski (*under review*). Forest inspectorates are divisions of state forest administration


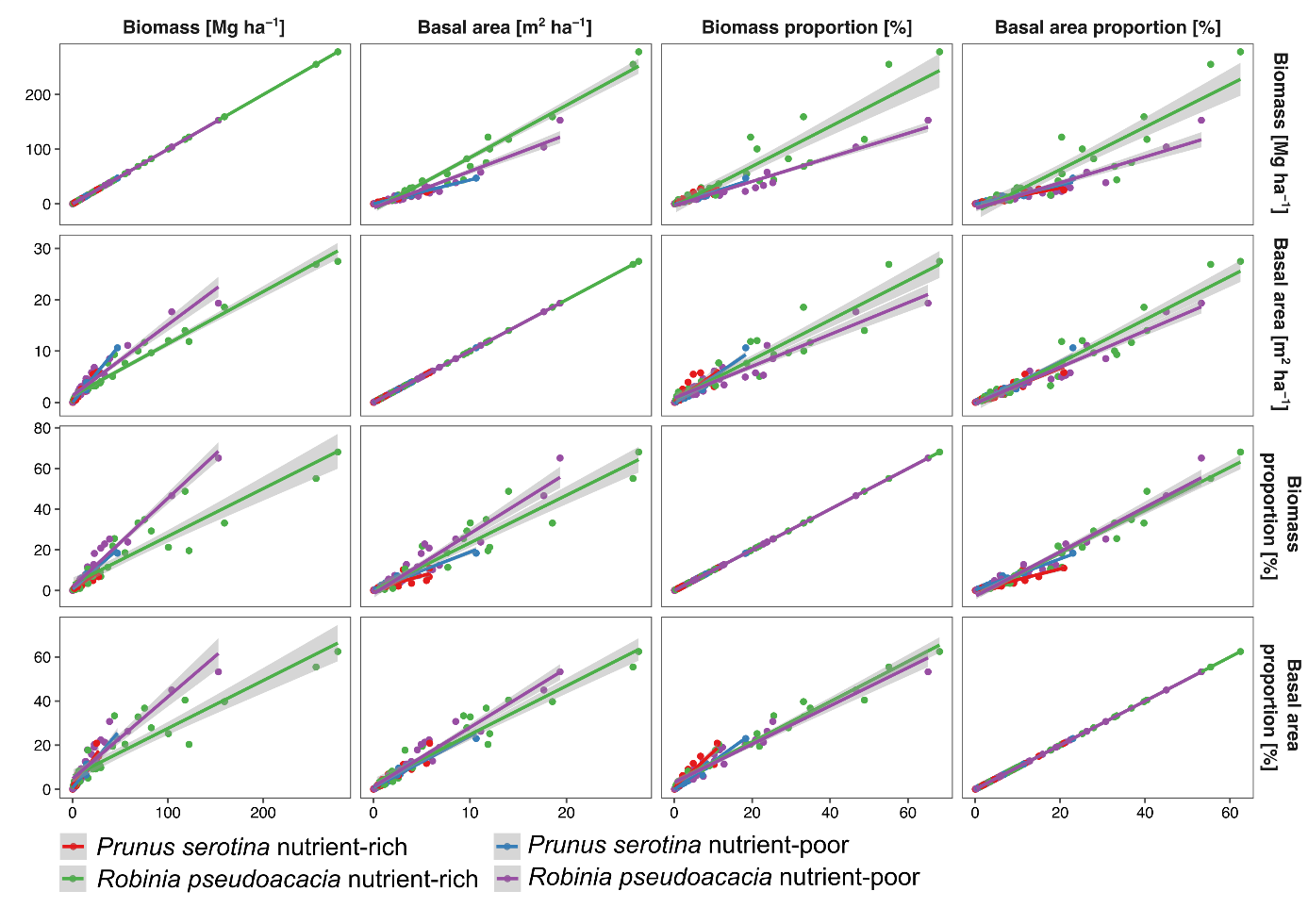


**Fig. S2.** Relationships between basal area, proportion of basal area in total stand basal area, aboveground biomass, and proportion in total stand aboveground biomass for studied invasive species in each type of habitats. For correlation coefficients see **Table S5**.

**Table S1.** List of species recorded in the study plots and their frequency (related to the 160 study plots).

| **Species** | **Frequency (%)** |
| --- | --- |
| *Brachythecium salebrosum* | 58.8 |
| *Hypnum cupressiforme* | 46.3 |
| *Pleurozium schreberi* | 45.6 |
| *Pseudoscleropodium purum* | 31.9 |
| *Dicranum polysetum* | 20.6 |
| *Polytrichum formosum* | 20.0 |
| *Dicranum scoparium* | 18.8 |
| *Plagiomnium affine* | 13.8 |
| *Atrichum undulatum* | 6.9 |
| *Leucobryum glaucum* | 2.5 |
| *Pohlia nutans* | 1.9 |
| *Rhytidiadelphus squarrosus* | 1.9 |
| *Hylocomium splendens* | 1.3 |
| *Brachythecium albicans* | 0.6 |
| *Cirriphyllum piliferum* | 0.6 |
| *Dicranella heteromalla* | 0.6 |
| *Eurhynchium angustirete* | 0.6 |
| *Plagiomnium undulatum* | 0.6 |
| *Rhizomium punctatum* | 0.6 |

**Table S2.** Details of Non-metric MultiDimensional Scaling of bryophyte species composition and statistics of fitting invader biomass as a passive environmental vector.

| **Variant** | **n** | **NMDS stress value** | **Predictor** | **NMDS1** | **NMDS2** | **r^2^** | **Pr(>r)** |
| --- | --- | --- | --- | --- | --- | --- | --- |
| *Prunus serotina*, nutrient-poor sites | 47 | 0.141 | Invader biomass | -0.911 | -0.413 | 0.019 | 0.673 |
| *Prunus serotina*, nutrient-rich sites | 48 | 0.098 | Invader biomass | -0.996 | 0.087 | 0.013 | 0.820 |
| *Robinia pseudoacacia*, nutrient-poor sites | 48 | 0.140 | Invader biomass | -0.753 | 0.6574 | 0.155 | 0.031 |
| *Robinia pseudoacacia*, nutrient-rich sites | 48 | 0.038 | Invader biomass | -0.418 | -0.910 | 0.156 | 0.119 |

Stress is a level of disagreement between 2-D configuration and dissimilarity values predicted from the regression, stress <0.2 is considered to be fair, <0.1 as good, while <0.05 as very good fit of NMDS.

**Table S3.** Results of Threshold Indicator Taxa Analysis for *P. serotina* on nutrient-rich sites (species with purity and reliability >0.95 bolded).

| **Variant** | **Species** | **ienv.cp** | **zenv.cp** | **freq** | **maxgrp** | **IndVal** | **obsiv.prob** | **zscore** | **purity** | **reliability** | **z.median** | **filter** |
| --- | --- | --- | --- | --- | --- | --- | --- | --- | --- | --- | --- | --- |
| *P. serotina* | *Brachythecium salebrosum* | 0.0 | 0.0 | 36 | 2 | 59.72 | 0.10 | 1.56 | 0.892 | 0.714 | 2.55 | 0 |
| Nutrient-poor sites | ***Dicranum polysetum*** | 1.5 | 1.5 | 25 | 1 | 75.78 | 0.00 | 6.88 | 1.000 | 1.000 | 7.28 | 1 |
|  | *Dicranum scoparium* | 0.8 | 0.8 | 24 | 1 | 48.52 | 0.03 | 2.56 | 0.922 | 0.820 | 3.36 | 0 |
|  | *Hypnum cupressiforme* | 0.7 | 0.7 | 22 | 1 | 51.95 | 0.00 | 3.68 | 0.894 | 0.902 | 4.20 | 0 |
|  | *Plagiomnium affine* | 2.3 | 2.3 | 5 | 2 | 31.43 | 0.05 | 3.13 | 0.828 | 0.604 | 3.43 | 0 |
|  | ***Pleurozium schreberi*** | 2.6 | 1.5 | 45 | 1 | 63.92 | 0.00 | 3.80 | 0.980 | 0.962 | 4.44 | 1 |
|  | *Polytrichum formosum* | 2.6 | 1.8 | 11 | 2 | 24.67 | 0.12 | 1.13 | 0.642 | 0.446 | 1.91 | 0 |
|  | ***Pseudoscleropodium purum*** | 0.0 | 0.6 | 35 | 2 | 68.35 | 0.00 | 4.65 | 0.978 | 0.976 | 5.01 | 2 |
| *P. serotina* | *Atrichum undulatum* | 2.7 | 0.0 | 7 | 2 | 20.59 | 0.10 | 1.70 | 0.828 | 0.588 | 2.45 | 0 |
| Nutrient-rich sites | *Brachythecium salebrosum* | 0.0 | 0.0 | 21 | 2 | 53.96 | 0.02 | 3.10 | 0.922 | 0.808 | 3.13 | 0 |
|  | *Hypnum cupressiforme* | 2.2 | 1.1 | 21 | 1 | 36.39 | 0.16 | 1.13 | 0.572 | 0.614 | 2.26 | 0 |
|  | *Polytrichum formosum* | 2.0 | 2.0 | 11 | 1 | 25.65 | 0.18 | 1.12 | 0.650 | 0.420 | 1.91 | 0 |
| *R. pseudoacacia* | *Brachythecium salebrosum* | 0.0 | 0.0 | 35 | 2 | 54.75 | 0.14 | 1.15 | 0.524 | 0.582 | 2.25 | 0 |
| Nutrient-poor sites | ***Dicranum polysetum*** | 0.0 | 0.9 | 19 | 1 | 61.78 | 0.00 | 5.31 | 0.986 | 0.970 | 6.01 | 1 |
|  | ***Dicranum scoparium*** | 0.9 | 0.9 | 14 | 1 | 64.78 | 0.00 | 7.32 | 1.000 | 1.000 | 7.52 | 1 |
|  | *Hypnum cupressiforme* | 0.0 | 0.0 | 29 | 2 | 46.28 | 0.20 | 0.55 | 0.318 | 0.450 | 1.90 | 0 |
|  | *Leucobryum glaucum* | 0.0 | 0.0 | 4 | 1 | 18.66 | 0.06 | 2.17 | 0.938 | 0.620 | 3.13 | 0 |
|  | *Plagiomnium affine* | 3.2 | 2.2 | 11 | 2 | 38.7 | 0.01 | 3.94 | 0.992 | 0.920 | 4.79 | 0 |
|  | ***Pleurozium schreberi*** | 0.0 | 0.0 | 40 | 1 | 79.7 | 0.00 | 5.27 | 0.998 | 0.994 | 5.24 | 1 |
|  | *Polytrichum formosum* | 2.3 | 2.3 | 7 | 2 | 14.55 | 0.34 | 0.19 | 0.486 | 0.460 | 2.04 | 0 |
|  | *Pseudoscleropodium purum* | 0.4 | 0.4 | 21 | 1 | 37.22 | 0.13 | 1.08 | 0.690 | 0.568 | 2.26 | 0 |
| *R. pseudoacacia* | *Brachythecium salebrosum* | 2.4 | 2.4 | 12 | 2 | 31.78 | 0.07 | 1.71 | 0.828 | 0.586 | 2.32 | 0 |
| Nutrient-rich sites | *Hypnum cupressiforme* | 4.1 | 0.0 | 18 | 1 | 40.35 | 0.12 | 1.27 | 0.778 | 0.566 | 2.12 | 0 |
|  | *Plagiomnium affine* | 0.0 | 0.0 | 4 | 1 | 17.49 | 0.15 | 0.92 | 0.556 | 0.216 | 1.34 | 0 |
|  | *Polytrichum formosum* | 4.3 | 4.3 | 9 | 2 | 30.12 | 0.13 | 1.10 | 0.630 | 0.592 | 2.57 | 0 |

**ienv.cp** ─ environmental change point for each taxon based on IndVal maximum; **zenv.cp** ─ environmental change point for each taxon based on z maximum; **freq** ─ number of non-zero abundance values per taxon; **maxgrp** ─ 1 if z- (negative response); 2 if z+ (positive response); **IndVal** ─ Dufrene and Legendre 1997 IndVal statistic, scaled 0-100%; **zscore** ─ IndVal 𝑧 score; **purity** ─ proportion of replicates matching observed **maxgrp** assignment; reliability ─ proportion of replicate obsiv.prob values < = 0.05; **z.median** ─ median score magnitude across all bootstrap replicates; **filter** ─ logical (if >0) indicating whether each taxa met purity and reliability criteria, value indicates maxgrp assignment. Abbreviations from Baker et al. (2020).

**Table S4**. Allometric equations determining the biomass of studied invasive tree species. Equations adopted were established for habitat conditions similar to those of this study. Abbreviations: DBH – diameter at breast height; CF - correction factor to reverse transformation of log-log models. Biomass components: AB – total aboveground biomass (ABW+FL), ABW – aboveground woody biomass, FL – foliage biomass.

| **Species** | **Biomass component** | **Unit** | **Source** | **R^2^** | **N** | **DBH min [cm]** | **DBH max [cm]** | | **Formula** | **a** | | **b** | | **CF** | |
| --- | --- | --- | --- | --- | --- | --- | --- | --- | --- | --- | --- | --- | --- | --- | --- |
| *Prunus serotina* | ABW | kg | Forrester et al. 2017 | 0.987 | 99 | 0 | | 50 | ln(Y)=ln(a)+b*ln(D), CF | | -1.09680 | | 2.09200 | | 1.02629 |
| >15 cm DBH | FL | kg | Forrester et al. 2017 | 0.545 | 99 | 0 | | 50 | ln(Y)=ln(a)+b*ln(D), CF | | -4.10580 | | 1.32120 | | 0.95641 |
| *Prunus serotina* | AB | kg | Jagodziński, unpbl. | 0.974 | 50 | 0 | | 15 | Y=a*D^b | | 0.84778 | | 1.44607 | | NA |
| <15 cm DBH | FL | kg | Jagodziński, unpbl. | 0.937 | 50 | 0 | | 15 | Y=a*D^b | | 0.04973 | | 1.86895 | | NA |
| *Robinia pseudoacacia* >10 cm DBH | AB | kg | Zasada 2017 | NA | 22 | 7 | | 46 | Y=a*D^b | | 0.00030 | | 2.51800 | | NA |
| *Robinia pseudoacacia* | ABW | kg | Forrester et al. 2017 | 0.923 | 165 | 0 | | 24 | ln(Y)=ln(a)+b*ln(D), CF | | -2.53440 | | 2.45980 | | 1.02110 |
| <10 cm DBH | FL | kg | Forrester et al. 2017 | 0.936 | 99 | 0 | | 24 | ln(Y)=ln(a)+b*ln(D), CF | | -2.79850 | | 1.12780 | | 1.02069 |

**Table S5.** Pearson’s correlation coefficients (r) for pairs of variables assessed for each pair of invasive species studied and habitat types.

| **Site and invader** | **Pair of variables** | **r** | **p** |
| --- | --- | --- | --- |
| Prunus serotina, nutrient-poor sites | Biomass-basal area | 0.993 | <0.001 |
| Prunus serotina, nutrient-rich sites | Biomass-basal area | 0.979 | <0.001 |
| Robinia pseudoacacia, nutrient-poor sites | Biomass-basal area | 0.961 | <0.001 |
| Robinia pseudoacacia, nutrient-rich sites | Biomass-basal area | 0.985 | <0.001 |
| Prunus serotina, nutrient-poor sites | Biomass-proportion in biomass | 0.975 | <0.001 |
| Prunus serotina, nutrient-rich sites | Biomass-proportion in biomass | 0.878 | <0.001 |
| Robinia pseudoacacia, nutrient-poor sites | Biomass-proportion in biomass | 0.980 | <0.001 |
| Robinia pseudoacacia, nutrient-rich sites | Biomass-proportion in biomass | 0.922 | <0.001 |
| Prunus serotina, nutrient-poor sites | Biomass-proportion in basal area | 0.961 | <0.001 |
| Prunus serotina, nutrient-rich sites | Biomass-proportion in basal area | 0.945 | <0.001 |
| Robinia pseudoacacia, nutrient-poor sites | Biomass-proportion in basal area | 0.936 | <0.001 |
| Robinia pseudoacacia, nutrient-rich sites | Biomass-proportion in basal area | 0.916 | <0.001 |
| Prunus serotina, nutrient-poor sites | Basal area - proportion in biomass | 0.975 | <0.001 |
| Prunus serotina, nutrient-rich sites | Basal area - proportion in biomass | 0.828 | <0.001 |
| Robinia pseudoacacia, nutrient-poor sites | Basal area - proportion in biomass | 0.957 | <0.001 |
| Robinia pseudoacacia, nutrient-rich sites | Basal area - proportion in biomass | 0.949 | <0.001 |
| Prunus serotina, nutrient-poor sites | Basal area - proportion in basal area | 0.972 | <0.001 |
| Prunus serotina, nutrient-rich sites | Basal area - proportion in basal area | 0.953 | <0.001 |
| Robinia pseudoacacia, nutrient-poor sites | Basal area - proportion in basal area | 0.974 | <0.001 |
| Robinia pseudoacacia, nutrient-rich sites | Basal area - proportion in basal area | 0.962 | <0.001 |
| Prunus serotina, nutrient-poor sites | Proportion in biomass-proportion in basal area | 0.990 | <0.001 |
| Prunus serotina, nutrient-rich sites | Proportion in biomass-proportion in basal area | 0.928 | <0.001 |
| Robinia pseudoacacia, nutrient-poor sites | Proportion in biomass-proportion in basal area | 0.974 | <0.001 |
| Robinia pseudoacacia, nutrient-rich sites | Proportion in biomass-proportion in basal area | 0.983 | <0.001 |
